# Supplementary figures and images for: Role of Phyllanthus niruri on the modulation of stress and immune responses in Nile tilapia, Oreochromis niloticus
Source: PLoS One. 2024 Oct 17;19(10):e0309793. doi: 10.1371/journal.pone.0309793 (PMC11486368; doi:10.1371/journal.pone.0309793)

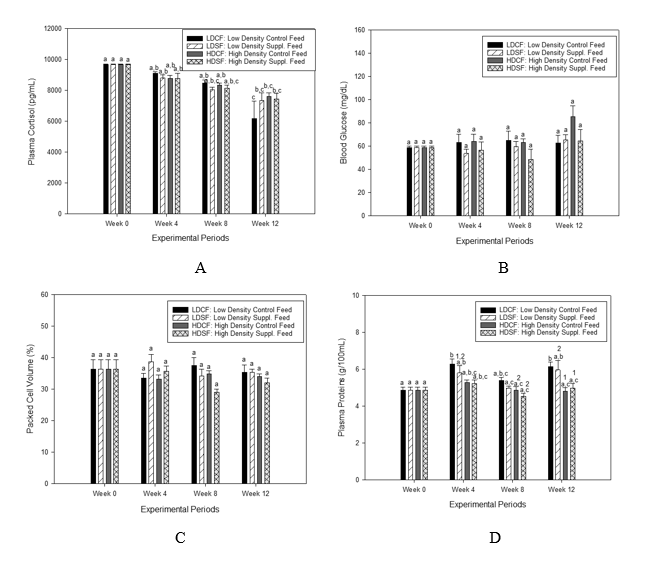

Supplement: S1 Fig — Data presented as means ± SEM, and N = 6 fish/group. Different letters and numbers indicate significant difference among different groups (One-way ANOVA, Tukey test, P<0.05). (TIF) [file pone.0309793.s001.tif]

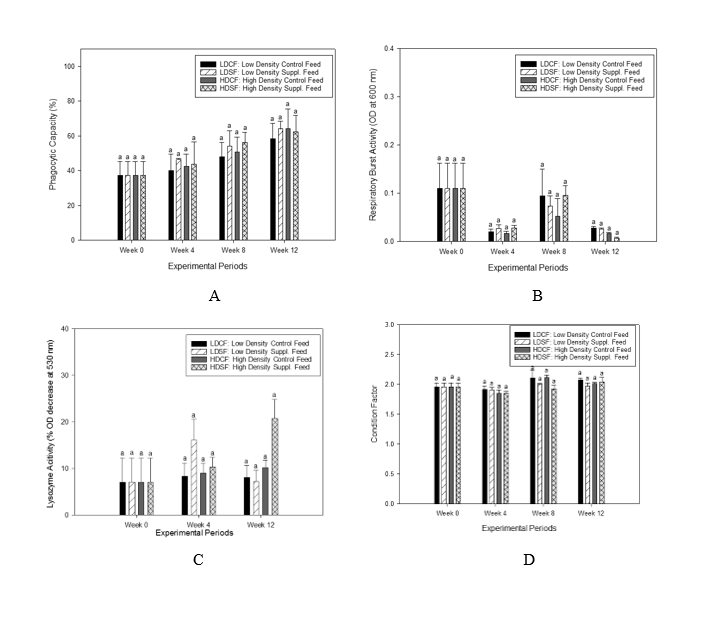

Supplement: S2 Fig — Data presented as means ± SEM, and N = 6 fish/group. Different letters indicate significant difference among different groups (One-way ANOVA, Tukey test, P<0.05). (TIF) [file pone.0309793.s002.tif]
