# Supplementary material for: Role of Phyllanthus niruri on the modulation of stress and immune responses in Nile tilapia, Oreochromis niloticus
Source: PLoS One. 2024 Oct 17;19(10):e0309793. doi: 10.1371/journal.pone.0309793 (PMC11486368; doi:10.1371/journal.pone.0309793)
Supplement: Attachment — Submitted filename: Response to revierwers.docx [file pone.0309793.s004.docx]

**Response to the reviewer’s comments**

**Ref: PONE-D-24-21421**

**Title: Role of *Phyllanthus niruri* on the modulation of stress and immune responses in Nile tilapia, *Oreochromis niloticus*.**

We would like to thank the reviewer for the valuable comments/suggestions.

We have incorporated all the corrections as suggested. (Please see the revised manuscript with track change).

Line 22: ‘reduce stress’ change to ‘minimize oxidative stress’
Line 24: ‘for Nile tilapia’ change to ‘in Nile tilapia, Oreochromis niloticus’
Line 35: include ‘generating’ after food
Line 40: Include ‘https://doi.org/10.1080/10641260500320845’
Line 48, 61, 63, 64: ‘induces reduction’ to change to ‘stimulate to decline’
Line 67: ‘require a long time to develop a vaccine’ change to ‘but it require to prepare vaccine long time’
Line 68: include ‘https://doi.org/10.1016/j.fsi.2010.02.026; <https://doi.org/10.1016/j.aquaculture.2010.09.011>’
Line 71: ‘within the hours’ change to ‘within short time’
Line 74, 76: include ‘ <https://doi.org/10.1016/j.aquaculture.2011.03.039>; <https://doi.org/10.1016/j.aquaculture.2011.07.022>’
Line 79: ‘options’ change to ‘measure’
Line 80: ‘option’ change to ‘alternative’
Line 83: include ‘and used in aquaculture practice’ after available
Line 95: no research has been done to investigate the effects of PN in the physiological and immunological responses of Nile tilapia, Oreochromis niloticus, but some other fish of the same and mentioned in this Ms:
<https://www.ncbi.nlm.nih.gov/pmc/articles/PMC5090155/pdf/ijvr-17-200.pdf>
<https://docs.lib.purdue.edu/dissertations/AAI10182448/>
<https://sdiopr.s3.ap-south-1.amazonaws.com/doc/Rev_AJFAR_77263_Jor_A.pdf>
<https://www.fisheriesjournal.com/archives/2017/vol5issue1/PartE/4-6-63-494.pdf>
<https://jchr.org/index.php/JCHR/article/view/1721>
<https://www.fisheriesjournal.com/archives/2023/vol11issue1/PartA/11-1-8-767.pdf>
Line 80: Here provide Water quality PH , dissolved oxygen, ammonia, and temperature levels..
Line 152: ‘1000 rpms for 5 mins’ change to ‘1000 rpm for 5 min’
Line 159, 176: ‘minutes’ change to ‘min’
Line 201: ‘Low-Density Control Feed (LDCF)’ change to ‘LDCF’
Line 204: ‘High-Density Control Feed (HDCF)’ change to ‘HDCF’
Line 217: ‘Low-Density Control Feed (LDCF) and Low-Density Supplemented Feed (LDSF)’ change to ‘LDCF and LDSF’
Line 219: Include ‘PN” before supplemented
Line 222: ‘the High-Density Control Feed (HDCF) and High-Density Supplemented Feed (HDSF)’ change to ‘HDCF and HDSF’
Line 259: ‘different’ change to ‘the’
Line 281, 301, 305, 308: ‘Phyllanthus niruri (P. niruri)’ change to ‘PN’
Line 282, 284, 287, 318: ‘P. niruri’ change to ‘PN’
Line 310: Related citation include

We thank the reviewer and the editor for their positive comments.
